# Laser-induced breakdown spectroscopy (LIBS) coupled with machine learning for classification of paddy soils from non-granary cultivation systems

**DOI:** 10.1038/s41598-026-56519-0

**Published:** 2026-06-08

**Authors:** Zubaidah Yusop, Khairul Fikri Tamrin

**Affiliations:** 1 Department of Mechanical and Manufacturing Engineering, Faculty of Engineering, Universiti Malaysia Sarawak (UNIMAS), Kota Samarahan, Sarawak 94300 Malaysia; 2 Faculty of Plantation and Agrotechnology, Universiti Teknologi MARA (UiTM), Cawangan Sarawak, Jalan Meranek, Kota Samarahan, Sarawak 94300 Malaysia; 3 Bio-Innovation and Biomedical Engineering Centre, Universiti Malaysia Sarawak (UNIMAS), Kota Samarahan, Sarawak 94300 Malaysia

**Keywords:** Laser-induced breakdown spectroscopy, Spectroscopy, Paddy soil, Principal component analysis, Support vector machine, Ecology, Ecology, Environmental sciences

## Abstract

Soil nutrients variability plays a crucial role in paddy growth and yield. Insufficient information on soil nutrient content can lead to inefficient fertilizers application, resulting in either nutrient deficiency or excess in the soil. This study investigates the potential of laser-induced breakdown spectroscopy (LIBS) combined with machine learning for rapid classification of paddy soils from non-granary cultivation systems in Sarawak, Malaysia. Soil samples from irrigated lowland, rainfed lowland, and upland areas were analysed using conventional physicochemical methods and LIBS to evaluate variability in soil properties and spectral characteristics. The LIBS spectra revealed distinct multi-elemental signatures dominated by Ca, Fe, K, and N, while P exhibited weak emission intensity due to both low concentration and intrinsic plasma emission limitations. Principal Component Analysis (PCA) reduced spectral dimensionality, with the first four components explaining 74.38% of the total variance. However, PCA score plots showed substantial overlap among soil groups, indicating limited separability using unsupervised analysis. To address this, a supervised classification approach using Support Vector Machine (SVM) was implemented. The PCA-SVM model achieved an average classification accuracy of 76.42 ± 15.17% under repeated sample-level hold-out validation, which improved to 87.33 ± 12.80% using Leave-One-Sample-Out Cross-Validation (LOSOCV). These results demonstrate that, although intrinsic spectral differences among paddy cultivation categories are subtle, the integration of LIBS with machine learning facilitates effective extraction of discriminative spectral patterns. This study highlights the potential applicability of LIBS as a rapid, multi-element analytical approach for preliminary classification of non-granary paddy cultivation systems, particularly in heterogeneous and resource-limited agricultural environments.

## Introduction

Paddy, or *Oryza sativa*, is the primary staple food for more than half of the world’s population. Due to its economic important, paddy is considered as a strategically important crop for Malaysia covering approximately 647,859 hectares involving more than 322,830 farmers nationwide^1^. About 64% of the total cultivation area is located within the 12 designated granary systems across the country, while the remaining area falls under the non-granary systems^1^. The state of Sarawak ranks as the fourth largest paddy cultivation area in the country with more than 61,342 hectares, after Kedah, Perak, and Kelantan^2^. With more than 98% of the cultivation areas are located in non-granary areas, Sarawak recorded the lowest yield with an average of 1.926 tonnes per hectare, which is considerably lower than the 5.0 tonnes per hectare target set under the National Agriculture Policy^2^. This can be attributed to limited modern infrastructure, lack of proper irrigation systems, and variability in soil properties, highlighting the importance of understanding soil variability for improved agricultural practices.

Soil variability plays a crucial role in influencing soil nutrient availability, and subsequently paddy productivity. Therefore, it is essential to obtain relatively accurate information on soil nutrient content and to employ reliable methods for assessing soil variability to support effective fertilization management strategies. This ensures that paddy crops receive sufficient nutrients according to soil condition and growth stages. Conventional methods for soil analysis such as atomic absorption spectroscopy (AAS) and inductively coupled plasma (ICP) provide an accurate measurement of soil properties. However, these techniques require extensive sample preparation and longer analytical times due to chemical extraction processes. For instance, soil nutrient analysis by AAS is inherently time-consuming and is limited to detecting a single element at a time^3^. Although ICP can detect multiple elements simultaneously, it still requires specific sample preparation and skilled laboratory operators^4^. For these reasons, these analytical techniques may be less suitable for rapid, large-scale soil assessment in paddy cultivation.

In contrast, laser-induced breakdown spectroscopy (LIBS) has been recognized as rapid analytical technique that capable of providing multi-element spectral information with minimal sample preparation without requiring time-consuming chemical procedures. Previous studies have demonstrated the application of LIB in agriculture and soil analysis, particularly for nutrient quantification and determination of soil properties. For example, Li et al^5^. demonstrated that LIBS can simultaneously identify total N, K, and exchangeable Ca in paddy soils. Wei et al^6^. utilized LIBS coupled with random forest algorithm to quantify the amount of ferrous sulphate fertilizer synthetically added to agricultural soil and the corresponding soil pH. Sha et al^7^. further demonstrated the capability of LIBS combined with support vector regression to quantitatively determine concentrations of N, P, and K elements in compound fertilizer. In another study, Wang et al^8^. demonstrated the potential of LIBS with an extreme learning machine to track the accumulation of cadmium in paddy roots at different growth stages.

This study advances the current state of knowledge by addressing a critical gap in the application of LIBS for soil classification under heterogeneous, low-input agricultural systems. Unlike most existing studies that focus on controlled or granary-based environments, this work systematically investigates the spectral characteristics of paddy soils from non-granary paddy cultivation systems in Sarawak, which are inherently variable and associated with significantly lower yields. The novelty lies not only in the underexplored study domain but also in the development of a robust, sample-level validation framework that integrates repeated hold-out and Leave-One-Sample-Out Cross-Validation (LOSOCV) thereby mitigating overfitting and data leakage. Furthermore, this study uniquely combines LIBS spectral analysis with conventional physicochemical soil characterization to enable a more interpretable and reliable classification of soil variability. These contributions highlight the potential applicability of LIBS for rapid soil assessment in resource-constrained paddy cultivation systems, where conventional laboratory-based soil analysis may be less accessible or time-consuming. Nevertheless, further validation using larger and geographically independent datasets, including field-based measurements, is necessary before broader practical implementation can be established.

## Materials and methods

**Fig. 1:**
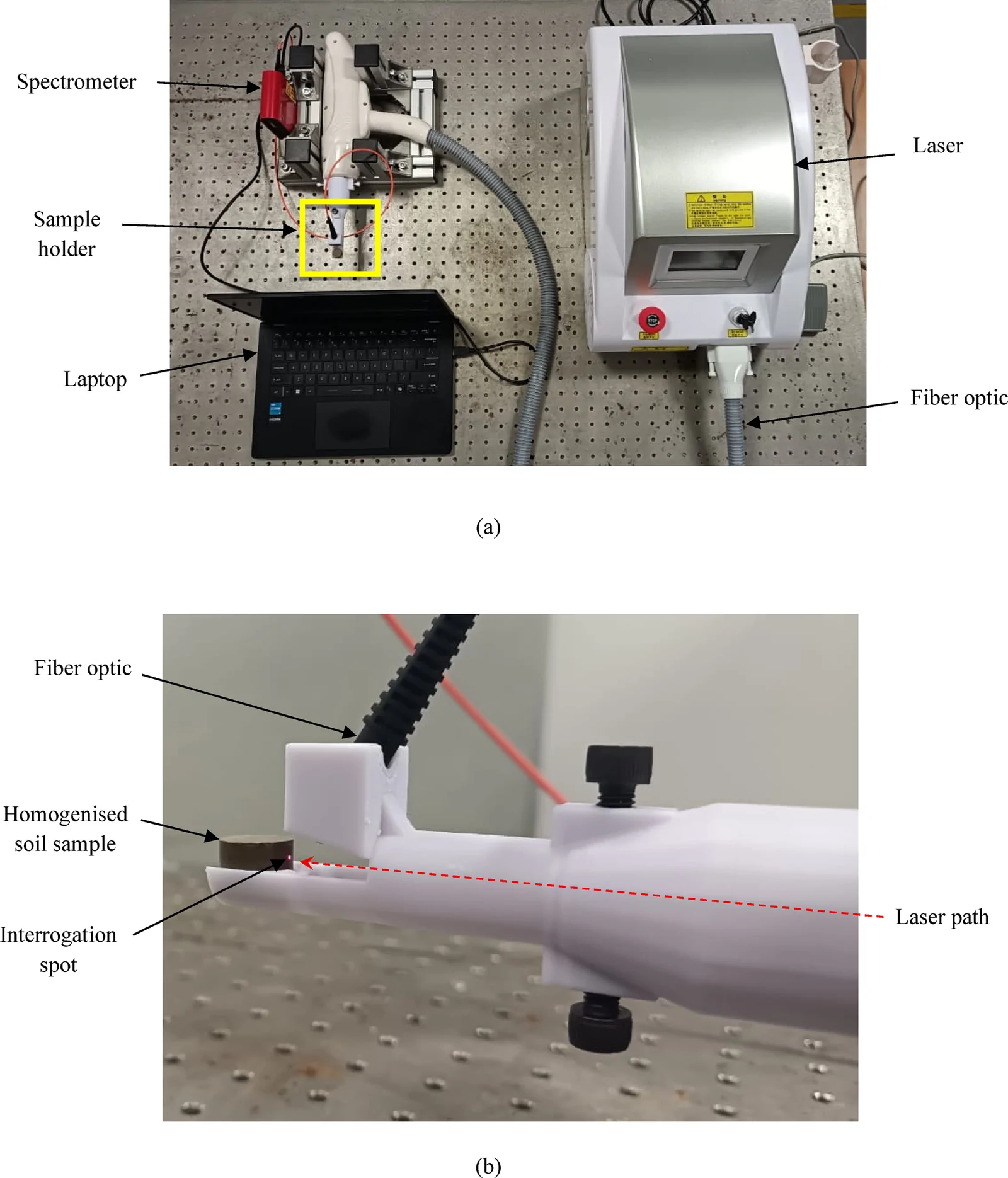
(**a**) The developed LIBS system for determining soil elements, (**b**) Side view of the sample holder containing a homogenised soil sample.

Figure 1 shows the LIBS system architecture for detecting the presence of soil elements, consisting of a solid-state laser, a spectrometer, a control system, and an optical system. The laser used in these experiments was a Nd: YAG laser operated in Q-switch mode at a fundamental wavelength of 1064 nm, with a pulse energy of 300 mJ, a pulse width of 10 ns, and a repetition rate of 1 Hz. The role of the laser is to initiate breakdown by focusing a high-energy laser beam onto the sample surface to ablate the sample material, heat it, and form a plasma plume that emits light^9^. The emitted light which contains information about the sample composition (presence of atomic lines) and parameters of the induced plasma, is captured by a spectrometer (Model CCS200/M, Thorlabs Inc.) to form a spectrum during plasma cooling. This spectrum can then be used to detect and quantify the presence of soil elements in paddy soil.

### Study location

The study area is classified as a non-granary paddy cultivation region. This research was conducted at three (3) different paddy cultivation areas in Sarawak, Malaysia: an irrigated lowland area, a rainfed lowland area, and an upland area. All study sites fall under the non-granary paddy cultivation system and have been used for paddy cultivation for more than five years. The irrigated lowland area is situated within the Sekuduk Chupak Paddy Scheme in Siburan. The rainfed lowland area is located in the Samarahan Division, while the upland paddy cultivation area is located at Gunung Tama in Serian. In general, the main cropping season for paddy cultivation in Sarawak occurs between November and February, while the off-season takes place from May to August. The rainfed lowland and upland cultivation areas are cultivated only during the main cropping season, whereas the irrigated lowland area supports paddy cultivation in both seasons. To minimize the influence of local weather and other external factors on the collected samples, all samples were obtained between April and May 2025, after the main cropping season harvest.

### Soil sampling

Soil samples were collected using an auger at a depth of approximately 20 cm from the soil surface to represent the active root zone of cultivated soil. A total of 15 soil samples were collected, consisting of five soil samples from each cultivation category, as summarized in Table 1. The positional data for each sampling point were georeferenced (latitude and longitude) using a conventional GPS device, while the slope and elevation for the upland area were recorded using a calibrated clinometer application. The collected soil samples were stored in zip-lock plastic bags, properly labelled, and transported to the laboratory for further analysis.

**Table 1:** Location of soil sampling.

Type of cultivation area	Sample point ID	GPS point at soil sampling points	Slope (^o^)/elevation (m)
Irrigated Lowland (IL)	IL-1	1°14’25.7"N 110°25’51.6"E	N/A
	IL-2	1°14’25.1"N 110°25’54.7"E	N/A
	IL-3	1°14’32.6"N 110°25’55.5"E	N/A
	IL-4	1°14’31.1"N 110°25’52.9"E	N/A
	IL-5	1°14’29.5"N 110°25’53.5"E	N/A
Rainfed Lowland (RL)	RL-1	1°28’54.7"N 110°28’09.8"E	N/A
	RL-2	1°28’55.3"N 110°28’09.1"E	N/A
	RL-3	1°28’54.5"N 110°28’08.8"E	N/A
	RL-4	1°28’53.4"N 110°28’08.7"E	N/A
	RL-5	1°28’52.6"N 110°28’08.5"E	N/A
Upland (UP)	UP-1	1°02’08.6"N 110°28’03.1"E	24.9^°^/189.89 m
	UP-2	1°02’08.6"N 110°28’04.0"E	29.2^°^/198.83 m
	UP-3	1°02’07.9"N 110°28’05.3"E	15.7^°^/208.81 m
	UP-4	1°02’08.0"N 110°28’05.7"E	27.4^°^/213.11 m
	UP-5	1°02’08.4"N 110°28’04.7"E	15.4^°^/204.49 m

### Soil sample preparation

**Fig. 2:**
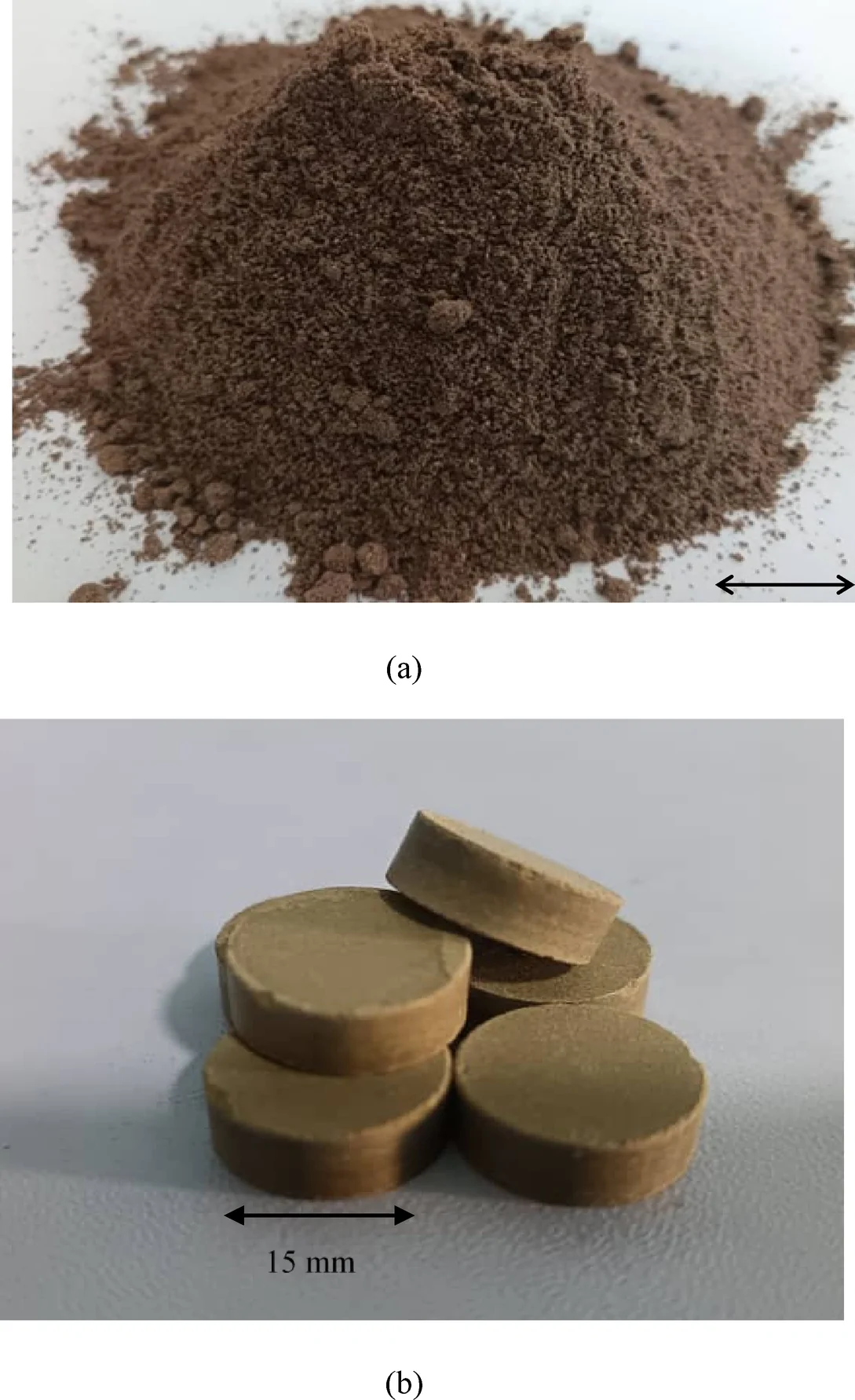
(**a**) Photos of pulverized soil, and (**b**) its pelletized samples obtained from lowland irrigated area.

The soil samples were air-dried naturally at room temperature (approximately 31.8^°^C) until a constant weight was achieved. The drying duration varied depending on the initial moisture content and typically ranged from 5 to 7 days. The drying process was conducted under ambient laboratory conditions without controlled humidity. However, all samples were dried under identical environmental conditions to ensure consistency. Air humidity was not continuously monitored during the drying process. After drying, the samples were crushed and pulverized into fine powder using a mechanical soil grinder, as shown in Fig. 2a. The powdered soil was then sieved through a 2 mm mesh to ensure particle size uniformity and to reduce sample heterogeneity. This homogenization step is critical for improving the repeatability of both conventional soil analysis and LIBS measurements. The processed soil powders were subsequently divided into two portions: one for conventional physicochemical analysis and the other for LIBS analysis.

For LIBS analysis, the soil powders were pelletized to improve surface uniformity, density, and laser–sample interaction stability. Pelletization is widely recognized as an effective approach to enhance spectral reproducibility and reduce shot-to-shot variability in LIBS analysis^10^. The improved density, surface flatness, and reduced porosity of pelletized samples contribute to more stable plasma formation, higher emission intensity, and lower spectral noise, thereby enhancing overall analytical precision and measurement reliability^11^. A fixed mass of 2 g of soil powder^6^, without the use of any binder material^12^, was weighed for each pellet to avoid potential spectral interference and matrix effects introduced by binding agents. The weighed powder was placed into a stainless-steel cylindrical mould and compressed into pellets with a diameter of 15 mm and a thickness of 3 mm using a universal testing machine (Model: Shimadzu). A constant compressive force of 5 kN was applied for 1 min^7^ for all samples to ensure uniform compaction and consistent physical properties across all pellets. To ensure measurement reproducibility and account for within-sample variability, each of the collected soil sample was used to prepare five replicates’ pellets (Fig. 2b), resulting 75 pellets in total across three paddy cultivation categories (15 soil samples × 5 pellets per sample).

### Soil analysis by conventional methods

The prepared soil samples were analysed for pH, EC, total N, total P, total K, available P, and available K. Soil pH was determined using a soil to water ratio of 1:2.5. Approximately 10 g of soil sample was weighed and mixed with 25 mL of distilled water in a beaker. The soil-water mixture was shaken for 5 min on the mechanical shaker and then allowed to stand for at least 2 h. The pH of the soil was measured using a calibrated pH meter (Model: CHECKER1 by Hanna Instruments) in accordance with MS 2457: 2012.

Soil electrical conductivity (EC) was determined using a soil to water ratio of 1:5. Approximately 10 g of soil sample was weighed and mixed with 50 mL of distilled water in a beaker. The soil-water mixture was then shaken for 30 min using the mechanical shaker to achieve uniform mixing. The suspension was then measured for electrical conductivity using the calibrated EC meter (Model: EC20 by Apera Instruments) in accordance with MS 2458: 2012.

Total nitrogen (TN) was determined using the Kjeldahl digestion method. Approximately 2 g of soil sample was weighed and placed into a Kjeldahl digestion tube. Then, 10 mL of concentrated sulphuric acid was mixed with the sample and shaken for 30 min using a mechanical shaker. A blank solution of sulphuric acid was prepared concurrently. The digestion tube was initially placed on the hot plate at low temperature. As the digestion progressed, bubbling and white smoke were observed. Once the white smoke appeared, 2–3 g of digesting promoter (90% potassium sulphate and 10% copper sulphate) was added and the temperature was increased. After the digestion process was completed, the tubes were allowed to cool down and were diluted with 10 mL of distilled water. The solution was further diluted up to 100 mL with distilled water. The digested sample was then transferred into a Kjeldahl distillation unit. Approximately 10 mL of 4% boric acid solution was placed in a 100 mL Erlenmeyer flask to capture the liberated ammonia. After completion, the receiving flask was removed, and the condenser tube was rinsed with distilled water. Then, the titration was carried out using 0.02 N standard sulfuric acid (H₂SO₄) until the end point which was indicated by a change in colour from blue to pale red. The volume of acid used was recorded, and the total nitrogen content was calculated accordingly.

To determine total phosphorus, approximately 2 g of finely ground soil samples were weighed into a Kjeldahl flask, and a mixture of sulphuric acid (H₂SO₄) and perchloric acid (HClO₄) was added. The sample was digested for about 2 h under moderate heat until complete decomposition was achieved. After cooling, the digest was transferred into a 100 mL volumetric flask, diluted to volume with distilled water, and filtered using Whatman No. 2 filter paper. An aliquot of 10 mL of the digest was transferred into a 50 mL volumetric flask, followed by the addition of 10 mL ammonium molybdate solution and 5 mL ascorbic acid solution. The solution was diluted to volume and mixed thoroughly. After 5 min of colour development, the absorbance was measured using a UV–Vis spectrophotometer at 660 nm. Available phosphorus, representing plant-available phosphorus, was extracted using the Bray II solution extraction method followed by UV-Vis spectrophotometric analysis. Approximately 1 gram of prepared soil sample was weighed and placed into a centrifuge tube. Then 7.0 mL of Bray II extracting solution was added, and the tube was shaken for 60 s to facilitate phosphorus extraction. The samples were then centrifuged at 6000 rpm for 5 min. An aliquot of 0.50 mL of the supernatant was collected and placed into a clean cuvette, followed by the addition of 2.0 mL of ascorbic acid–molybdate reagent. The mixture was then allowed to stand for 30 min for colour development. Prior to analysis, the reagent blank was used to zero the UV–Vis spectrophotometer. The samples were then analysed using UV–Vis spectrophotometer at a wavelength of 880 nm.

For total potassium (TK) determination, approximately 1 gram of prepared soil sample was weighed and placed into a crucible and heated on a hot plate for 30 min to remove moisture. The sample was subsequently ashed in a furnace at 800 °C for 1 h to eliminate organic matter. After cooling, the ash was digested using 10 mL of concentrated hydrochloric acid (HCl). The digested solution was then filtered using filter paper into a 50mL volumetric flask and diluted to volume with distilled water. For available potassium (AK), 1 gram of prepared soil sample was weighed and placed into an extraction flask. Then 10 mL of 0.1 N hydrochloric acid (HCl) was added into the soil sample and agitated for 1 h at 210 rpm using a mechanical shaker. After shaking, the suspension was filtered using filter paper into a 50 mL volumetric flask and diluted to volume with distilled water before analysis. Both TK and AK were analysed using a flame photometer.

### LIBS spectral acquisition

LIBS measurements were conducted under atmospheric laboratory conditions. During spectral acquisition, laser shots were randomly distributed across the surfaces of the prepared pellets. Each laser spot was irradiated only once to avoid surface burning effects and signal distortion caused by repeated laser exposure at the same location. To further minimize shot overlap and maintain surface consistency, the pellets were rotated throughout the measurement process. For each soil sample, 30 LIBS spectra were acquired across the corresponding replicate pellets. This procedure was repeated for all soil samples representing irrigated lowland, rainfed lowland, and upland cultivation categories, generating a total dataset of 450 LIBS spectra.

### Analysis by machine learning

Machine learning-based analyses were performed using MATLAB R2017b for: (i) preliminary PCA to identify the main sources of variability in the soil spectral data and to explore clustering patterns among irrigated, rainfed and upland cultivation categories; and (ii) predictive classification to evaluate the discrimination performance of the LIBS spectra using PCA-SVM modelling under sample-level validation conditions.

i. Preliminary PCA analysis

Prior to PCA, the spectral data were standardized to zero-score normalisation (mean = 0, standard deviation = 1) to reduce potential bias in signal intensity and ensure that all variables contributed equally to multivariate analysis. Preliminary PCA was performed on all individual spectral data (450 spectra) to capture overall spectral variability and reduce the dimensionality of the large dataset into a smaller set of PCs while still maintaining significant patterns and trends of the data. The PCs, eigenvalues and eigenvectors, were obtained by computing the covariance matrix, where the eigenvalues represented the variance explained by each PC and the eigenvectors described the contribution of spectral variables to the corresponding principal components. The PCA score plot and scree plots were also generated to visualize the clustering patterns of the soil samples based on their similarities. To further interpret the spectral features contributing to variability, PCA loading plots were analysed by identifying wavelengths with high absolute loading values. These observed wavelengths were assigned to their corresponding elemental emission lines using reference data from the National Institute of Standards and Technology (NIST) atomic spectra database.

ii. Predictive classification of PCA-SVM analysis at sample level.

**Fig. 3:**
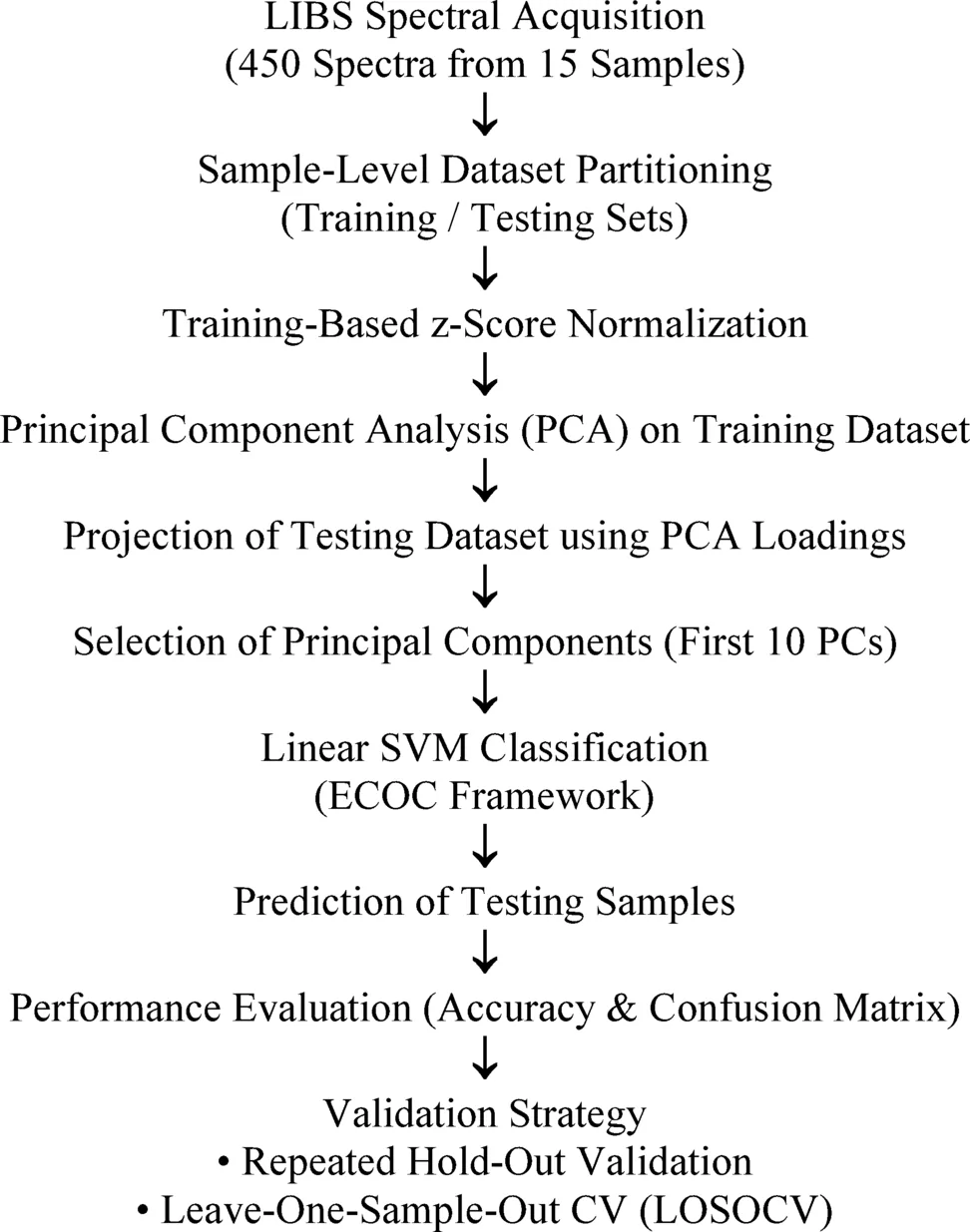
Schematic workflow of the leakage-free PCA-SVM predictive classification framework used for LIBS soil discrimination.

For predictive classification analysis, a separate PCA-SVM workflow was implemented using repeated hold-out validation at the sample level to evaluate the discrimination performance of the LIBS spectral dataset under independent validation conditions, as shown in Fig. 3. During each validation iteration, 70% of the samples were randomly assigned to the training subset, while the remaining 30% were reserved as a testing subset. All spectra originating from the respective soil sample were maintained exclusively within either the training or testing subset to prevent spectra derived from the same soil sample from appearing simultaneously in both subsets during model evaluation. The hold-out validation procedure was repeated ten (10) times using different random sample partitions to improve model robustness and reduce bias associated with random data partitioning.

To avoid preprocessing-related data leakage, sample-level partitioning was performed prior to normalization and PCA analysis. Within each validation iteration, z-score normalization parameters were calculated exclusively from the training dataset and subsequently applied to normalize the testing dataset using the same transformation parameters. PCA was then performed only using the normalized training spectra to reduce dimensionality and extract the dominant spectral variance. The resulting PCA loading coefficients obtained from the training dataset were subsequently applied to transform the testing dataset into the same PCA feature space.

The first 10 PCs representing the main source of variability of the dataset were selected as input variables for classification because they retained the majority of cumulative spectral variance while reducing data dimensionality and minimizing noise contributions. A linear Support Vector Machine (SVM) classifier based on the Error-Correcting Output Code (ECOC) framework was subsequently trained using the PCA-transformed training dataset and used to predict the class labels of the independent testing dataset. Classification performance was evaluated based on the average prediction accuracy and confusion matrix obtained across all repeated hold-out validation iterations.

## Results and discussion

### Soil texture characterization

**Fig. 4:**
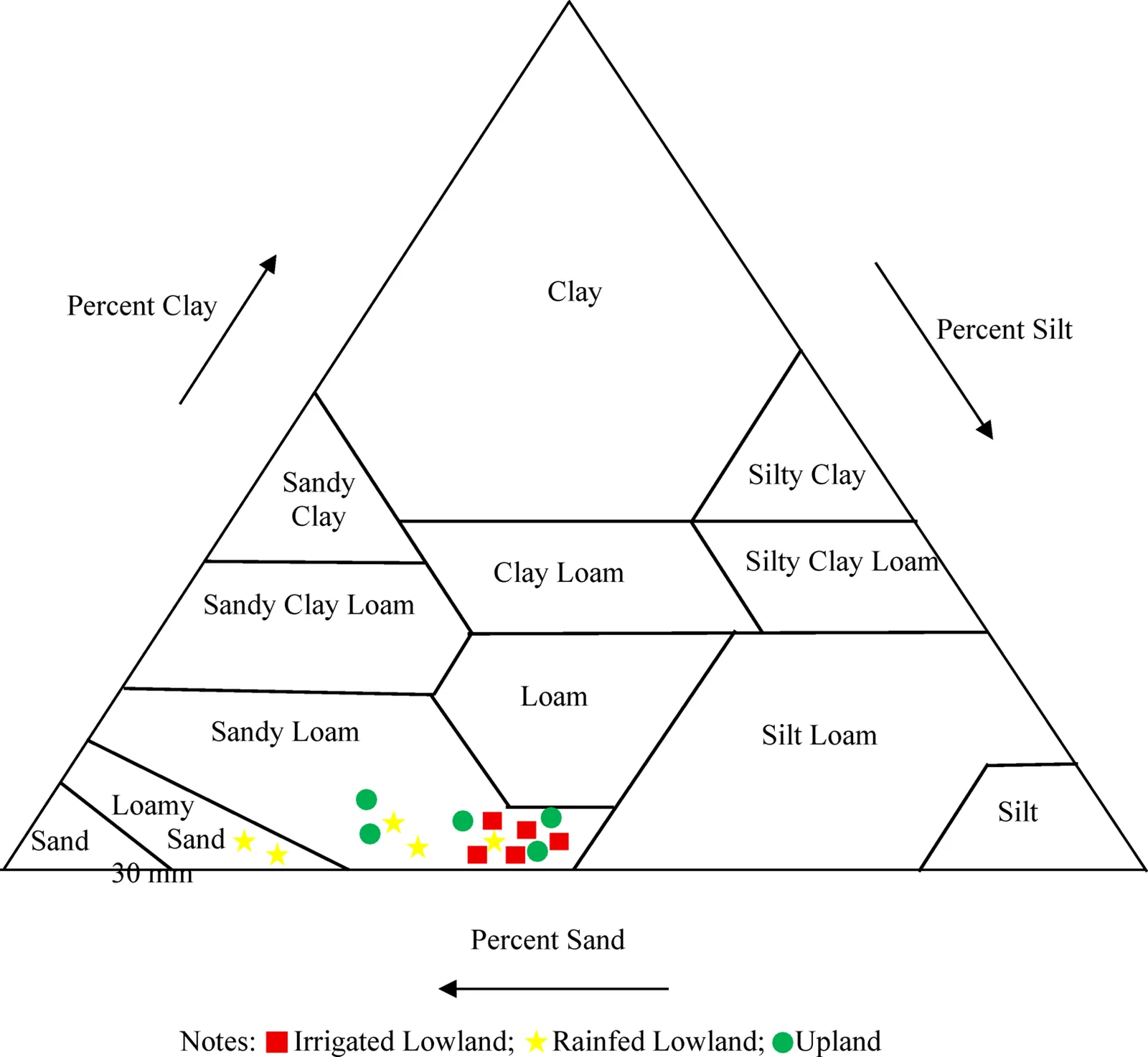
Mapping of the collected soil samples onto the USDA soil textural triangle.

Soil texture is one of the most commonly used criteria for soil classification. It is determined based on the relative proportion of sand, silt and clay in the soil^13^. According to United States Department of Agriculture (USDA) Soil Taxonomy, the soil textures of the three paddy cultivation areas in this study were classified as sandy loam and loamy sandy, as shown in Table 2^14^. Based on Table 2; Fig. 4, the majority of the sampling points were classified as sandy loam, whereas loamy sand was observed only in the lowland cultivation area (RL-4 and RL-5). These samples exhibited the highest sand content (73.91% to 83.33%), along with relatively low silt content (14.81% to 24.64%) and clay content (1.45% to 1.85%). This finding is consistent with the study by Shwetha and Varija^15^ who reported that paddy cultivation areas are often characterized by high sand content and relatively low silt and clay fractions.

Soil texture significantly influences both the physical and chemical properties of soil, including water-holding capacity, porosity, organic matter content, and nutrient availability^16^. The observed sandy loam and loamy sand textures indicate larger particle sizes and pore spaces, which can promote nutrient leaching. In contrast, finer-textured soils generally exhibit better aggregate stability, allowing for greater retention of organic carbon and total nitrogen^17^. Furthermore, previous studies have shown that variations in soil texture can directly affect nutrient availability in soils^4,13^.

**Table 2:** Soil texture in study area.

Types of cultivation area	Sample point ID	Percentage (%)			Soil texture
		Sand	Silt	Clay	
Irrigated lowland (IL)	IL-1	56.72	40.30	2.98	Sandy Loam
	IL-2	57.41	38.89	3.70	Sandy Loam
	IL-3	53.57	42.86	3.57	Sandy Loam
	IL-4	55.56	36.11	8.33	Sandy Loam
	IL-5	52.94	42.65	4.41	Sandy Loam
Rainfed lowland (RL)	RL-1	55.55	41.67	2.78	Sandy Loam
	RL-2	61.91	33.33	4.76	Sandy Loam
	RL-3	66.67	31.03	2.30	Sandy Loam
	RL-4	73.91	24.64	1.45	Loamy Sand
	RL-5	83.33	14.81	1.85	Loamy Sand
Upland (UP)	UP-1	68.11	26.09	5.80	Sandy Loam
	UP-2	59.65	38.60	1.75	Sandy Loam
	UP-3	68.77	26.15	5.08	Sandy Loam
	UP-4	53.97	44.44	1.59	Sandy Loam
	UP-5	52.38	46.02	1.60	Sandy Loam

### Soil chemical analysis of paddy soil using conventional methods

Geographical variation among paddy cultivation areas leads to measurable differences in soil physicochemical and elemental composition, even within the same region of Sarawak. These differences are primarily governed by variations in soil texture, irrigation regimes, fertilizer management, and site-specific agricultural practices. Table 3 summarizes the average soil nutrient properties for the three cultivation systems based on conventional soil analyses.

Soil pH is a critical parameter influencing nutrient solubility, microbial activity, and overall nutrient uptake efficiency. The soil pH values observed in this study ranged from 4.98 to 5.42, indicating generally acidic conditions across all sites. The upland cultivation area exhibited the lowest average pH (4.98), while both irrigated and rainfed lowland areas showed similar values (5.42). Although paddy can tolerate mildly acidic soils, optimal nutrient availability typically occurs at pH values above 5.0. Soils with pH values near or below this threshold may require liming to improve nutrient availability and microbial activity. Highly acidic conditions are known to adversely affect both chemical equilibria and biological processes in soils, thereby limiting crop productivity^18^.

EC which reflects the concentration of soluble ions in soil, showed clear variation among the cultivation systems. The irrigated lowland area recorded the highest average EC (178.74 µS/cm), followed by the upland (91.74 µS/cm) and rainfed lowland (65.64 µS/cm) areas. This trend is consistent with previous findings indicating that increased soil moisture enhances ionic mobility and accumulation, thereby elevating EC values^19^. The relatively high EC in the irrigated lowland system can be attributed to sustained water input and retention, which promote the accumulation of dissolved salts and nutrients. This occurs despite similar soil textures across the sites, suggesting that hydrological conditions play a dominant role in controlling EC.

Macronutrient distributions further highlight the influence of environmental and management factors. The upland area exhibited the highest total nitrogen content, followed by the rainfed lowland and irrigated lowland systems. The comparatively low nitrogen content in the irrigated lowland area can be explained by prolonged waterlogging conditions observed during sampling. Such conditions create anaerobic environments that suppress microbial activity and enhance nitrogen losses through denitrification and leaching. In addition, elevated EC levels may further inhibit microbial processes, thereby reducing nitrogen mineralization rates. In contrast, the higher nitrogen content in rainfed lowland and upland areas is likely associated with better soil aeration, which promotes microbial activity and nitrogen cycling. The practice of retaining crop residues after harvest further contributes to nitrogen availability through organic matter decomposition and mineralization. In the upland system, despite sloping terrain that may encourage runoff and erosion, enhanced aeration and active microbial processes appear to compensate for potential nitrogen losses.

The distribution of phosphorus reveals a more complex interaction between soil chemistry and topography. The rainfed lowland area recorded the highest total phosphorus content, followed by the upland and irrigated lowland areas. This accumulation is likely associated with repeated fertilizer applications, particularly in systems practicing crop rotation, leading to residual phosphorus buildup over time. However, a key observation is that the upland area, despite having relatively high total phosphorus, exhibited the lowest available phosphorus. This indicates strong phosphorus fixation, likely driven by acidic soil conditions (pH = 4.98) and the presence of iron (Fe) and aluminium oxides, which immobilize phosphorus in insoluble forms. As reported by Yigezu et al^18^., acidic soils promote phosphorus fixation and reduce its mobility and bioavailability. Furthermore, the sloping topography (15.4° – 29.2°) of the upland area enhances surface runoff and erosion, accelerating nutrient loss and limiting water retention. Reduced soil moisture further restricts phosphorus diffusion to the root zone. This is consistent with findings by Li et al^20^. who demonstrated that increased slope gradient intensifies runoff-driven nutrient loss, thereby reducing phosphorus availability. The combined effects of chemical fixation and physical transport processes ultimately explain the low phosphorus availability in the upland system.

Potassium dynamics also varied across the cultivation systems. The rainfed lowland area exhibited the highest total potassium content, followed by the irrigated lowland and upland areas. In the rainfed lowland system, this may be attributed to the deposition of potassium-rich sediments transported via rainfall and riverine processes, particularly from the nearby Batang Samarahan River. Additionally, soil mineral composition, especially clay content, plays a role in potassium retention through adsorption and fixation mechanisms. In the irrigated lowland area, elevated potassium levels may be influenced by geological factors, including weathering and erosion of upland parent rocks bearing minerals such as feldspar and mica^21^ from nearby highland regions such as Gunung Chupak, as well as sediment transport via irrigation systems. However, despite relatively high total potassium, elevated EC levels in this area may reduce potassium availability due to cation competition at exchange sites. Interestingly, the upland area, despite having the lowest total potassium, exhibited the highest available potassium. This suggests more active potassium cycling, likely facilitated by better soil aeration, coarser texture, and enhanced mineral weathering. These conditions promote the release of potassium from primary minerals and organic residues, increasing its availability for plant uptake.

It is noted that the observed variability in soil physicochemical properties across the three cultivation systems reflects the combined influence of hydrological conditions, soil chemistry, topography, and agricultural practices. These variations are expected to directly influence the elemental emission characteristics captured by LIBS, particularly through differences in elemental concentration, matrix effects, and plasma formation behavior. This relationship is further explored in the subsequent section.

**Table 3:** Conventional analysis for soil nutrients in three (3) different cultivation area of soil in Sarawak.

Types of cultivation area	Sample point ID	pH	EC (µS/cm)	Total *N* (%)	Total *P* (mg/kg)	Total K (mg/kg)	Available *P* (mg/kg)	Available K (mg/kg)
Irrigated lowland (IL)	IL-1	5.6	125.9	0.19	226	4051	16	55
	IL-2	5.2	251	0.12	218	2571	10	40
	IL-3	5.1	244	0.16	197	3175	12	29
	IL-4	5.8	163.8	0.15	184	3072	13	44
	IL-5	5.4	109	0.15	210	2934	9	35
Average		5.42	178.74	0.15	207	3160.6	12	40.6
Range		5.1–5.8	109–251	0.12–0.19	184–226	2571–4051	9–16	29–55
Rainfed lowland (RL)	RL-1	5.7	57.6	0.18	244	3425	76	117
	RL-2	5.6	42.7	0.22	301	3558	15	76
	RL-3	5.1	50.2	0.51	286	2800	20	107
	RL-4	5.4	80	0.47	259	3428	17	91
	RL-5	5.3	97.7	0.15	295	2927	9	77
Average		5.42	65.64	0.306	277	3227.6	27.4	93.6
Range		5.1–5.7	42.7–97.7	0.15–0.51	244–301	2800–3558	9–76	76–117
Upland (UP)	UP-1	5.3	66.5	0.26	230	812	1	198
	UP-2	5.0	56.4	0.26	255	936	1	152
	UP-3	5.0	81.7	0.40	206	1033	2	293
	UP-4	4.9	87.8	0.39	206	738	1	254
	UP-5	4.7	67.3	0.38	202	545	1	96
Average		4.98	71.94	0.338	219.8	812.8	1.2	198.6
Range		4.7–5.3	56.4–87.8	0.26–0.40	202–255	545–1033	1–2	96–293

### Elemental composition detected by LIBS

**Fig. 5:**
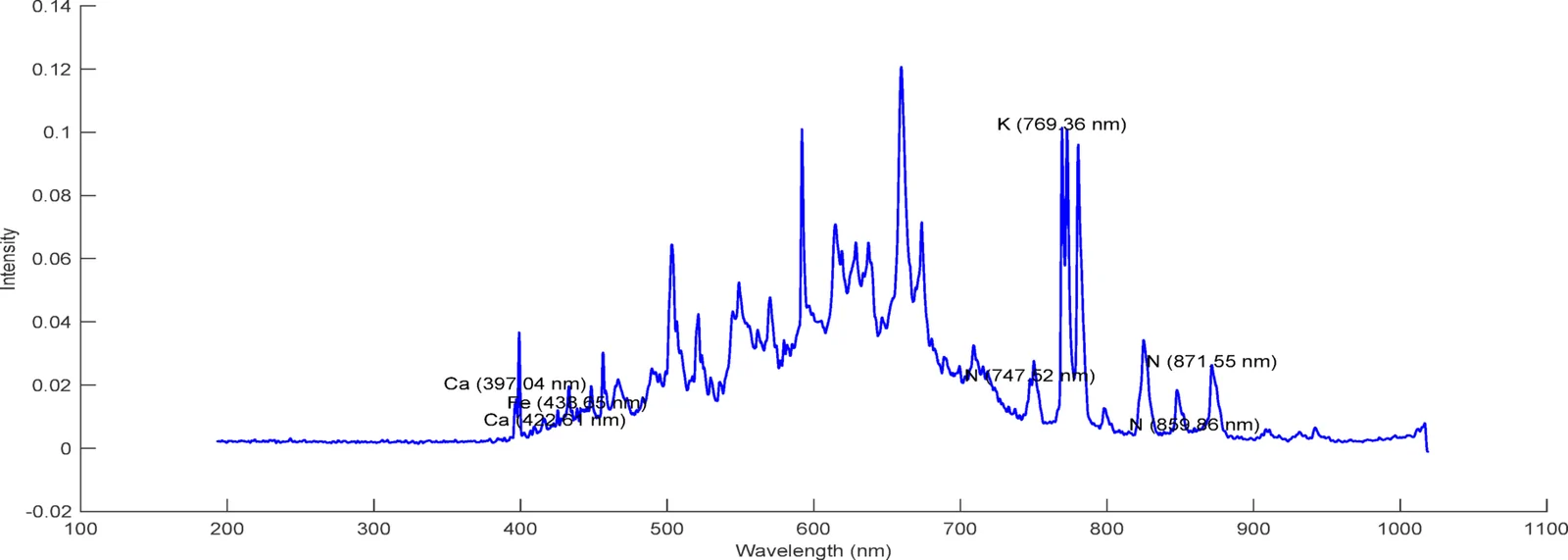
Sample of LIBS spectrum for irrigated lowland area showing some major elements.

Figure 5 shows the LIBS spectrum obtained from soil samples collected in the irrigated lowland paddy cultivation area, revealing multiple emission peaks associated with key soil elements, including calcium (Ca), Fe, K and N. These prominent spectral features demonstrate the capability of LIBS to capture the multi-elemental composition of complex soil matrices within a single acquisition. In contrast, the emission lines corresponding to P within the 213.5–255.5 nm region exhibit comparatively low intensities. This observation is consistent with the laboratory-determined total and available phosphorus concentrations (Table 3), suggesting that the weak spectral response is primarily due to the low phosphorus content in the soil. Furthermore, the intrinsically low emission efficiency of phosphorus in LIBS plasma, particularly in the deep ultraviolet region, further limits its detectability.

Despite this limitation, the overall spectral profile highlights a key advantage of LIBS, namely its ability to simultaneously detect multiple elements without the need for extensive sample preparation. This represents a substantial improvement over conventional analytical techniques, which often require separate digestion or extraction procedures for individual nutrients. As such, LIBS offers a rapid and versatile alternative for high-throughput elemental screening, while providing comprehensive compositional information beyond the capabilities of standard laboratory methods.

Importantly, the observed spectral variations are consistent with the differences in soil physicochemical properties discussed earlier, thereby reinforcing the reliability of LIBS in capturing underlying soil composition. Variations in emission intensities across different elements and cultivation systems indicate that each soil type possesses a distinct spectral signature. Although certain elements, such as phosphorus, produce relatively weak emission signals, their consistent relative variations across samples remain informative and contribute meaningfully to the overall spectral pattern.

These subtle yet systematic spectral differences formed the basis for multivariate analysis. PCA was employed to reduce spectral dimensionality while preserving the dominant sources of variance, thereby enabling visualization of clustering patterns within the dataset. Subsequently, SVM classification was applied to improve class separability by constructing optimal decision boundaries within the transformed feature space. The integration of LIBS with these multivariate techniques facilitated discrimination among the irrigated lowland, rainfed lowland, and upland cultivation categories despite the presence of relatively weak or overlapping elemental signals.

### Preliminary PCA analysis of LIBS spectra

**Fig. 6:**
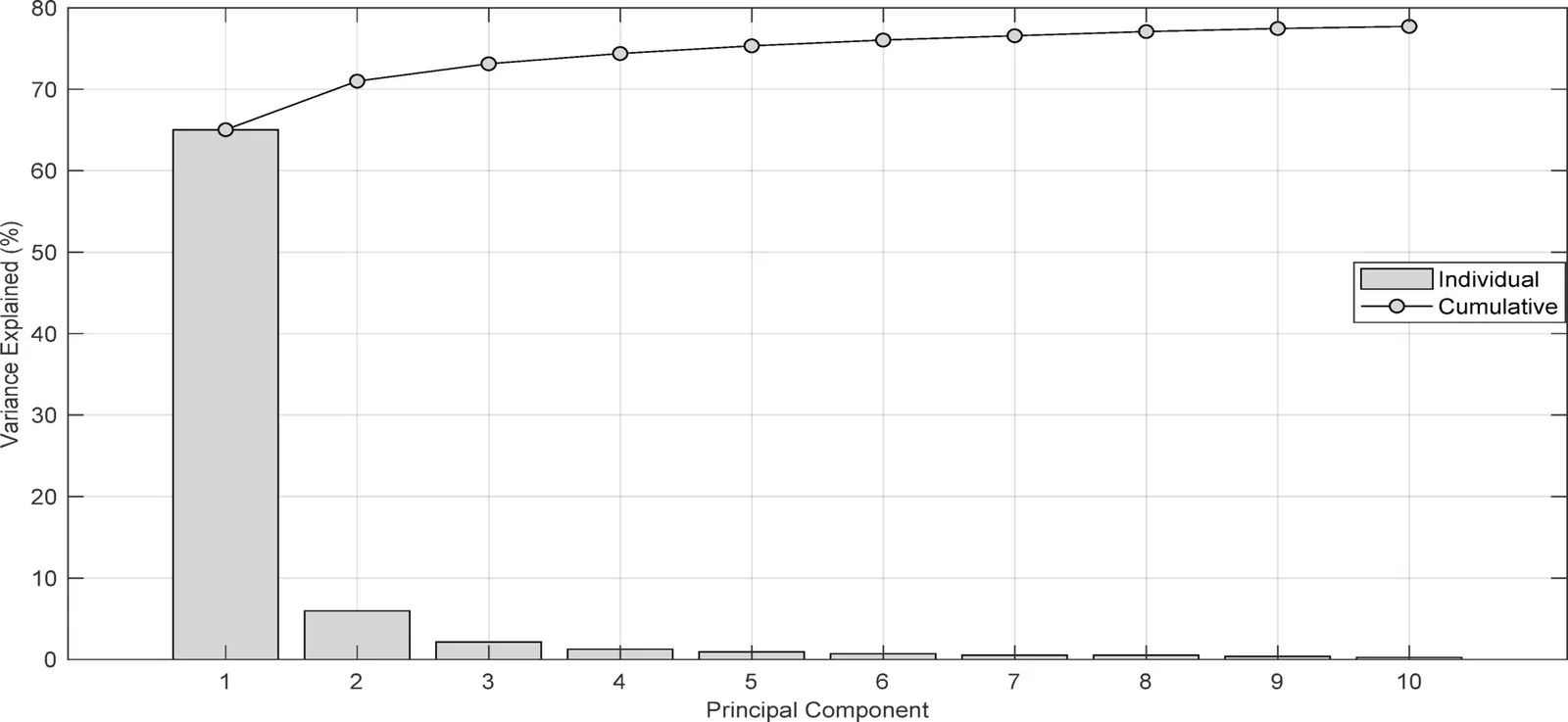
Scree Plot for 10 PCs.

The LIBS spectral data obtained from irrigated lowland, rainfed lowland, and upland cultivation areas were initially analysed using PCA to evaluate spectral variability and clustering behaviour among the three paddy soil groups. PCA was employed to reduce the high dimensionality of the LIBS spectra by extracting the principal components that capture the dominant sources of variance within the dataset. As summarized in Table 4; Fig. 6, a total of 10 principal components (PCs) were extracted, collectively accounting for 77.72% of the total variance in the dataset. However, only the first four PCs exhibited eigenvalues greater than 1, cumulatively explaining 74.38% of the variance. This indicates that the majority of meaningful spectral information is concentrated within the leading components, while the remaining PCs primarily capture noise and minor variations. Among these, PC1 exhibited the highest loading contribution (0.836), confirming its dominant role in representing the overall spectral variability. This suggests that the primary differences in elemental composition across the soil samples are effectively captured along the first principal component. The strong variance concentration within the first few PCs further justifies dimensionality reduction prior to classification, as it enhances computational efficiency while preserving the most discriminative features.

**Fig. 7:**
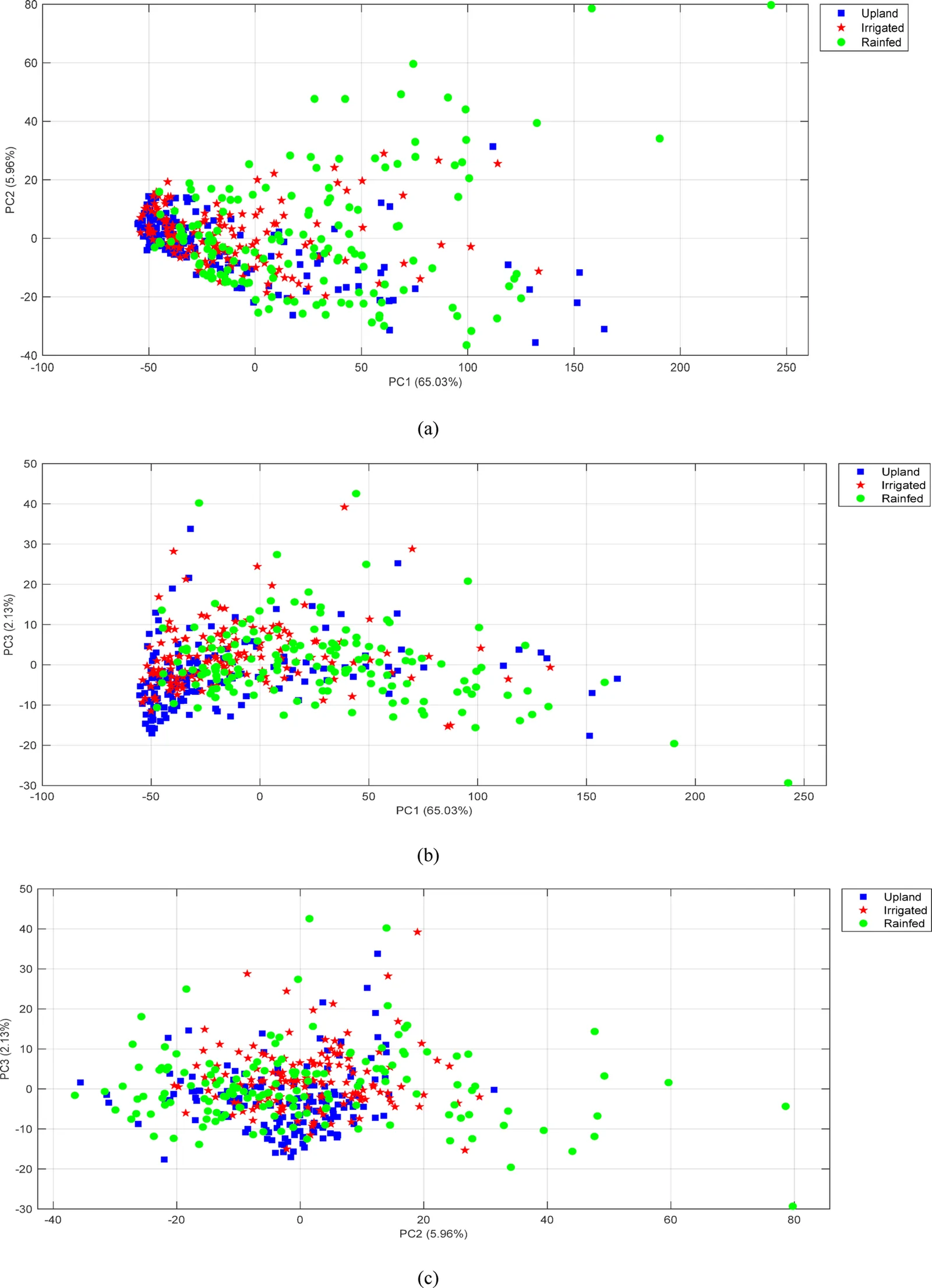
PCA score plots for (**a**) PC1 vs. PC2, (**b**) PC1 vs. PC3 **(c**) PC2 vs. PC3.

The PCA score plots (Fig. 7) reveal a substantial degree of overlap among the irrigated, rainfed, and upland soil groups, indicating that their LIBS spectral profiles are largely similar. PC1 alone explains a significant proportion of the total variance (65.03%), whereas PC2 and PC3 contribute comparatively smaller proportions (5.96% and 2.13%, respectively). The broader dispersion of rainfed lowland samples along PC1 suggests slightly higher heterogeneity in their elemental composition relative to the other soil groups. However, the absence of well-defined clusters in the PCA space indicates that the intrinsic spectral differences captured by LIBS are subtle and insufficient for clear discrimination using unsupervised analysis alone. This observation is consistent with the complex and overlapping nature of soil elemental compositions, particularly in agricultural systems with similar management practices and mineralogical characteristics.

**Table 4:** Principal components, eigenvalues, percent variance, and cumulative variance and weighted factor.

PC	Eigenvalue	Percent variance	Cumulative variance	Weighted factor
1	2371.7415	65.0327	65.0327	0.8368
2	217.4493	5.9624	70.9951	0.0767
3	77.7577	2.1321	73.1272	0.0274
4	45.8296	1.2566	74.3838	0.0162
5	34.3496	0.9419	75.3257	0.0121
6	25.9847	0.7125	76.0382	0.0092
7	19.3013	0.5292	76.5674	0.0068
8	18.6707	0.5119	77.0794	0.0066
9	13.7515	0.3771	77.4564	0.0049
10	9.4287	0.2585	77.715	0.0033

**Fig. 8:**
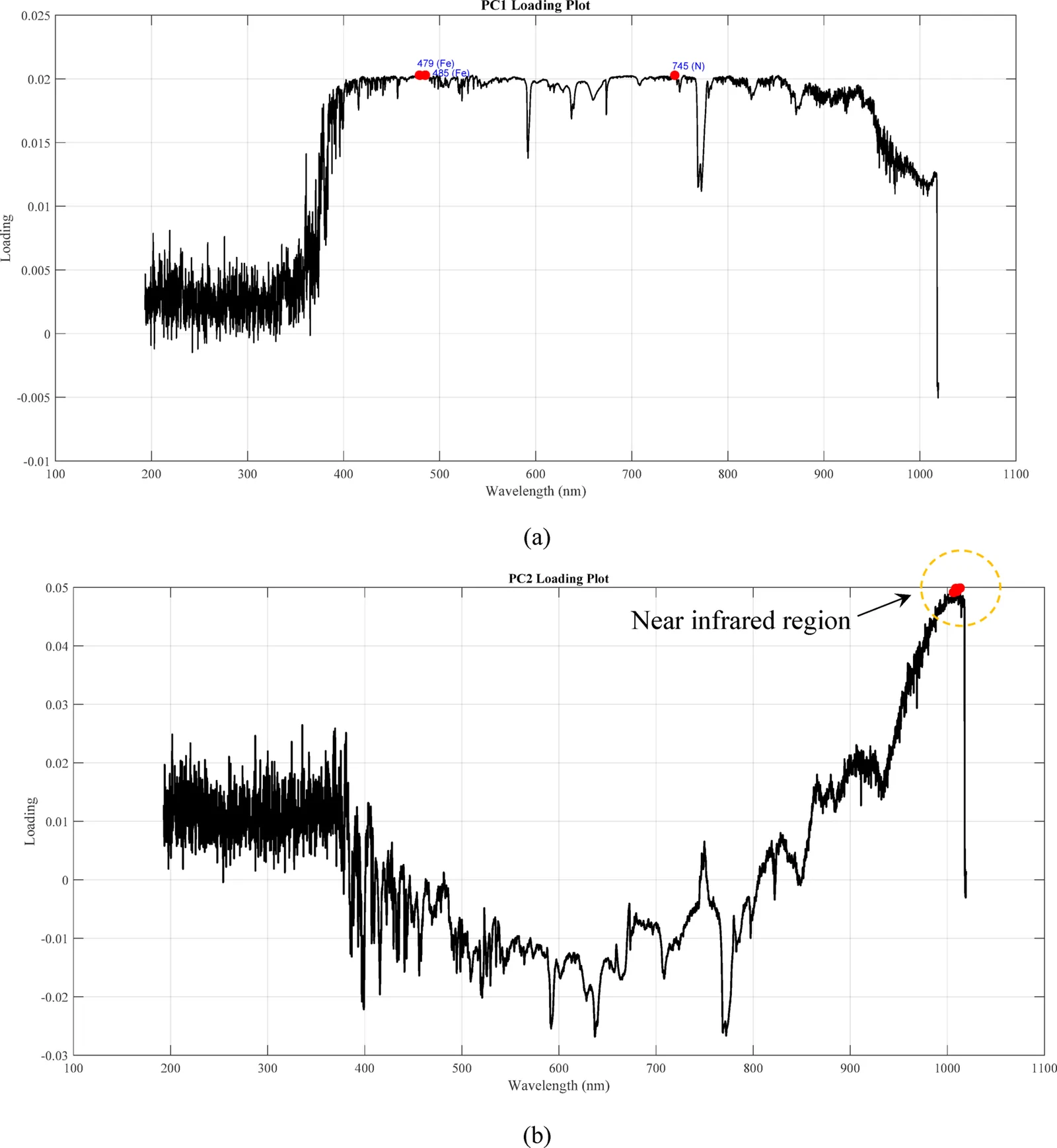
Loading plots for (**a**) PC1 and (**b**) PC2.

To further interpret the sources of variability identified by PCA, the loading plot of PC1 (Fig. 8) was examined to determine the key spectral regions contributing to class separation. The observed spectral features were assigned by comparison with reference emission lines reported in the NIST database. The results indicate that the dominant variability captured by PC1 arises from spectral features in the 479–485 nm region, which correspond to characteristic emission lines of Fe. This suggests that variations in soil mineral composition, particularly Fe-bearing minerals, play a major role in discriminating between soil classes. In addition, a weaker feature around 745 nm is tentatively attributed to N emission, although its lower intensity indicates a secondary contribution.

In contrast, PC2 is primarily associated with spectral features in the near-infrared region (~ 1006–1013 nm), which do not correspond to strong elemental emission lines in the NIST database. These features are therefore interpreted as matrix-related effects, likely reflecting variations in soil physicochemical properties such as texture and EC. This distinction between PC1 and PC2 highlights that PCA captures both elemental composition and matrix-driven variability in the LIBS spectra.

Further analysis of higher-order principal components provides additional insight into subtler sources of variation. PC3 shows contributions from Fe and magnesium (Mg), suggesting co-variation of mineral phases such as ferromagnesian components commonly present in soils. PC4 is dominated by infrared spectral features, reinforcing the influence of matrix effects rather than discrete elemental emissions. PC5 is associated with N, which may reflect variability in soil organic matter content, as N in soils is often linked to organic fractions. PC6 and PC7 are influenced by K and sodium (Na), respectively, indicating variability in exchangeable cations and soil fertility status. In particular, stronger K emission lines correspond to soils with higher exchangeable potassium content, while weaker P-related emissions are consistent with the low available P measured using conventional soil analysis. PCs 8 and 9 again show contributions from Fe, with PC9 additionally involving Ca and Na, suggesting mixed mineralogical and ionic influences. Differences in ionic elements (e.g., Ca, Mg, Na) may also reflect variations in EC, further contributing to class separation. Finally, PC10 represents a combination of multiple elements, indicating low-variance residual patterns where no single element dominates.

The progressive shift from strong, chemically interpretable signals in lower PCs (e.g., Fe in PC1) to mixed and weaker contributions in higher PCs reflects the hierarchical nature of PCA, where the most significant sources of variance are captured first, while subsequent components describe increasingly subtle and complex interactions among elemental composition and soil matrix properties. This interpretation suggests that the LIBS–PCA analysis was capable of capturing multiple sources of spectral variability associated with both geochemical and physicochemical properties of the soil samples. However, LIBS-PCA cannot differentiate the three different soil cultivation categories.

### Predictive PCA-SVM classification performance

**Fig. 9:**
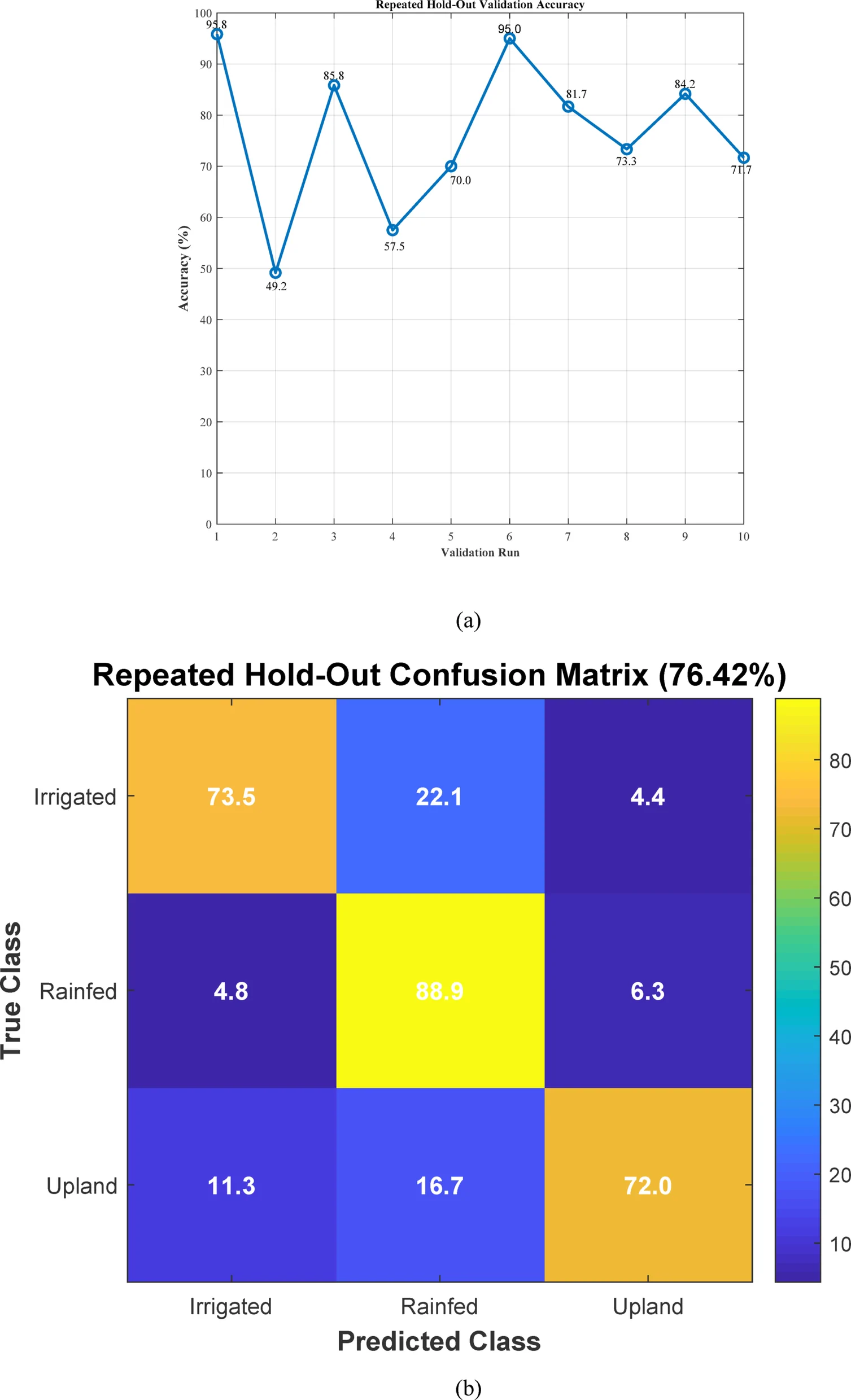
PCA-SVM classification model under repeated sample-level hold-out validation showing (**a**) repeated hold out validation accuracy and (**b**) averaged confusion matrix.

To address the limited class separation observed in the PCA analysis, a supervised classification approach using a linear SVM was implemented to exploit the subtle spectral variations that are not readily separable in the unsupervised PCA score space. In this study, the first ten PCs were selected as input features for the SVM model, as they preserve a substantial proportion of the total variance (77.72%) while retaining discriminative information that may be distributed across higher-order components. The integration of PCA with SVM facilitated dimensionality reduction and feature compression prior to classification, thereby reducing data complexity and improving computational efficiency. The PCA-SVM model achieved an average accuracy of 76.42 ± 15.17% under repeated sample-level hold-out validation (Fig. 9). While the relatively large standard deviation indicates sensitivity to different data partitions, this behaviour is expected given the limited number of independent soil samples included in the present study and highlights the importance of implementing leakage-free preprocessing and independent validation strategies when working with relatively small LIBS spectral datasets.

**Fig. 10:**
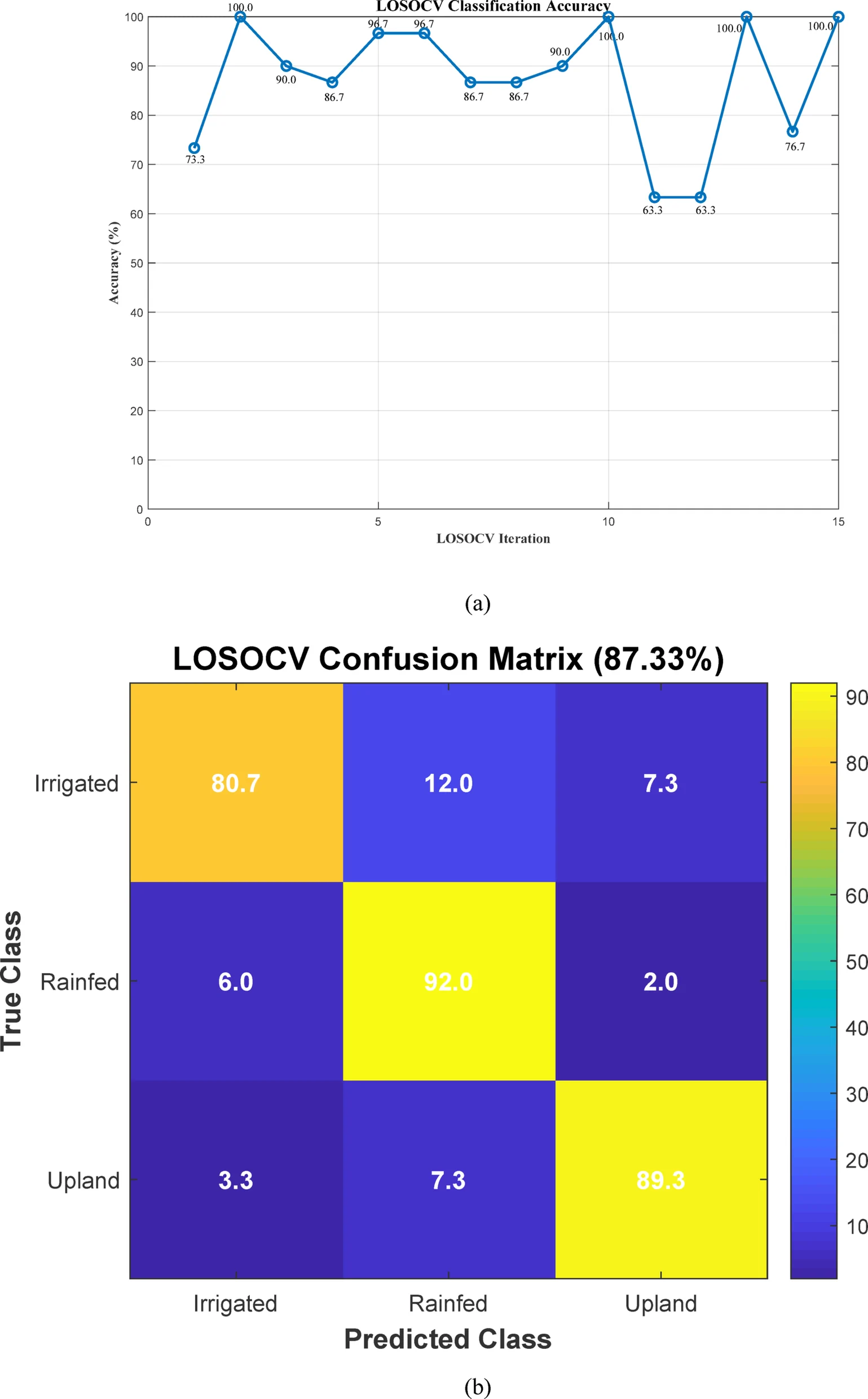
PCA-SVM classification performance evaluated using Leave-One-Sample-Out Cross-Validation (LOSOCV): (**a**) classification accuracy obtained across 15 LOSOCV iterations and (**b**) average confusion matrix.

To further evaluate model generalization under fully independent sample-level validation, LOSOCV was subsequently performed. The LOSOCV approach yielded an improved average classification accuracy of 87.33 ± 12.80% (Fig. 10), indicating that the PCA-SVM model was capable of extracting reproducible spectral patterns under fully independent sample level validation. The improved performance observed under LOSOCV is likely associated with the larger proportion of training samples utilized during each validation iteration, which is advantageous when working with relatively small datasets.

These results suggest that, although LIBS spectral differences among the cultivation category are not strongly separable in an unsupervised framework, the application of supervised machine learning enables effective extraction of subtle yet systematic variations. This highlights the potential of combining LIBS with advanced multivariate machine learning techniques for preliminary soil classification under controlled laboratory conditions, even in cases where spectral contrasts are inherently weak.

### Limitations of the study

Despite the encouraging performance of the proposed classification framework, several limitations should be acknowledged to provide a balanced interpretation of the results and to guide future work. First, the study is constrained by a relatively limited dataset, comprising 15 soil samples collected from three agricultural areas within a single state. This restricted spatial coverage may limit the generalisability of the developed models to broader geographical regions with different soil compositions and environmental conditions. A more extensive sampling campaign covering diverse soil types and locations would be necessary to further evaluate model reliability and external applicability.

Second, all measurements were performed on pelletised samples under controlled laboratory conditions. While this approach improves signal stability and reduces measurement variability, it does not fully represent real-world field conditions. In-situ LIBS measurements may introduce additional challenges such as surface heterogeneity, moisture variation, and environmental interferences, which were not addressed in this study. Therefore, the applicability of the current model to direct field deployment remains to be validated.

Third, model evaluation was performed using internal validation strategies, namely repeated hold-out (70:30 split) and Leave-One-Sample-Out-Cross-Validation (LOSOCV) at the sample level. Although spectra originating from the same soil sample were grouped together during dataset partitioning to minimize potential data leakage, both validation approaches were still based on the same overall dataset and do not involve external independent validation datasets. Consequently, the true generalisation performance of the classification model on unseen soil samples from different locations remains to be further investigated.

Finally, preliminary analysis using PCA revealed noticeable overlap between classes in the reduced feature space. This indicates that the intrinsic separability of the classes is not strongly pronounced and that classification performance relies on supervised learning algorithms to capture subtle spectral differences. This also suggests that further feature engineering or advanced modelling approaches may be required to enhance class discrimination. These limitations highlight important directions for future work, particularly in expanding the dataset, incorporating field-based measurements, and performing external validation using independent datasets to strengthen the broader applicability of the proposed LIBS–machine learning framework.

## Conclusion

Soil variability influences the nutrient availability and consequently affects the growth and productivity of paddy. This study demonstrated the capability of LIBS, combined with PCA and SVM, for the classification of paddy soils from non-granary cultivation areas in Sarawak. LIBS successfully captured multi-element spectral information across all samples, reflecting underlying differences in soil physicochemical properties. While PCA effectively reduced spectral dimensionality, the absence of distinct clustering in the score plots indicates that spectral differences among the soil groups are subtle and not readily separable using unsupervised methods alone. The application of a supervised linear SVM classifier significantly improved class discrimination, achieving an average accuracy of 76.42 ± 15.17% under repeated sample-level hold-out validation and of 87.33 ± 12.80% under LOSOCV. These findings confirm that SVM is capable of capturing complex, non-obvious spectral patterns that are not directly observable in reduced-dimensional space. Importantly, this study demonstrates that LIBS-based soil classification does not rely solely on strong individual elemental signals but rather on the collective spectral response, including subtle and low-intensity features. However, the study was limited by the relatively small number of independent soil samples and restricted spatial coverage. Future work should focus on expanding the dataset, incorporating additional soil types, and validating the model using independent field-scale data. Overall, the integration of LIBS with advanced multivariate analysis presents a promising potential for rapid soil characterization and preliminary soil discrimination in precision agriculture research.

## Data Availability

Data availability The LIBS spectral dataset generated and analyzed during the current study are publicly available in the Zenodo repository at: https://doi.org/10.5281/zenodo.20437121.
